# Synthesis of Fe_1-x_S Nanoparticles with Various Superstructures by a Simple Thermal Decomposition Route and Their Magnetic Properties

**DOI:** 10.3390/nano11061447

**Published:** 2021-05-30

**Authors:** Aleksandr A. Spivakov, Chun-Rong Lin, Yu-Chuan Chang, Ying-Zhen Chen

**Affiliations:** Department of Applied Physics, National Pingtung University, Pingtung County 90003, Taiwan; aleksandr.a.spivakov@gmail.com (A.A.S.); bek106006@nptu.edu.tw (Y.-C.C.); yingzhen0307@gmail.com (Y.-Z.C.)

**Keywords:** Fe_1-x_S nanoparticles, thermal decomposition, vacancy distribution, magnetic properties, synthesis conditions

## Abstract

Pyrrhotite nanoparticles with 5C and 3C superstructures were synthesized via a simple one-step thermal decomposition method in which hexadecylamine was used as a solvent at various reaction temperatures (T_R_). Structural analysis showed that at T_R_ = 360 °C, almost uniform in size and shape Fe_7_S_8_ nanoparticles with 3C superstructure are formed, and an increase in the reaction temperature leads to the formation of Fe_9_S_10_ nanoparticles (5C superstructure), herewith a significant increase in the size of nanoparticles is observed. High-temperature magnetic measurements in 5 repeated heating-cooling cycles revealed that after the first heating branch in the Fe_9_S_10_ samples, the λ—Peak transition disappears, and the magnetization has a Weiss-type behavior characteristic of the Fe_7_S_8_ sample. The change in the behavior of magnetization can be explained by the redistribution of iron vacancies, which changes the initial phase composition of nanoparticles.

## 1. Introduction

Natural iron sulfide minerals play an important role in geochemistry, environmental, microbiology, and marine systems [1,2,3,4], while synthesized iron sulfides have found applications in various fields [5,6,7,8,9]. There are a variety of iron sulfides, the main ones being pyrite (FeS_2_), pyrrhotite (Fe_1-x_S), mackinawite (FeS), and greigite (Fe_3_S_4_) [10]. Pyrrhotites have attracted great interest for a long time from both scientific [11,12,13] and practical points of view since they have potential practical applications in such areas as biomedicine, phase-change magnetic memory devices, water treatment, as an anode material of Li-ion batteries, and so on [14,15,16,17,18,19]. The pyrrhotite group with the general chemical formula Fe_1-x_S (0 ≤ x ≤ 0.125) includes several nonstoichiometric compounds, which are due to different concentrations of vacancies in iron atom sites [13]. Pyrrhotites have the hexagonal close-packed NiAs type structure with sulfur atoms in the hexagonal close packing, and iron atoms are located at the centers of the octahedra of the sulfur atoms; herewith, pyrrhotites demonstrate various superstructures due to the different ordering of Fe atoms and cationic vacancies [20,21]. Additionally, magnetic studies of the pyrrhotite group have shown that magnetic behavior is closely related to their composition and, at room temperature, can be divided into three regions [20,22,23,24,25,26,27,28]: (1) 0 ≤ x ≤ 0.05—Stoichiometric or almost stoichiometric antiferromagnetic troilite (space group $P\bar{6}2c$) with 2C superstructure (lattice parameters a = $\sqrt{3}A$, c = 2C, where A and C—The axes of the NiAs subcell); (2) 0.11 < x ≤ 0.125—Monoclinic or hexagonal ferrimagnetic pyrrhotite with 4C superstructure (1A, $2\sqrt{3}A$, 4C), which can be considered as a derivative of the NiAs structure of FeS by the removal of one-eighth iron atoms. As a result, a structure is formed in which close-packed layers of sulfur atoms alternate with layers fully occupied with Fe atoms and layers of Fe positions with vacancies. The ferrimagnetic behavior is associated with an uncompensated moment arising from the presence of vacancies in alternating layers; (3) 0.05 < x ≤ 0.11—Hexagonal pyrrhotites in which “λ—Peak transition” between an antiferromagnetic and ferrimagnetic states takes place. In this range, a set of so-called “NC” pyrrhotite superstructures (a = 2A; c = NC, 5 ≤ N ≤ 11) is formed, and some pyrrhotites in the range may represent ordered phases with defined compositions, such as Fe_9_S_10_ (5C), Fe_10_S_11_ (11C), and Fe_11_S_12_ (6C), however, partial ordering in these systems may also occur. Such superstructures are described in terms of stacking of fully occupied and ordered defective iron layers normal to the c-axis, and each such structure is related with a regular succession of such layers, corresponding to an integral supercell multiplicity N (or non-integral N in the case of deviations from the ordered succession, which leads to an incommensurate c-axis).

One of the reasons limiting the development and study of iron sulfides for practical application is their relatively complex synthesis, therefore, various methods for the synthesis of iron sulfides have been described in the literature, such as electrospinning [29], the toluene-thermal process [30], freeze-drying process [31], hot injection chemical synthesis [8], and so on. Iron sulfides can usually be obtained from their respective minerals via mining and separation. Among the synthesis methods, one of the most common is the high-temperature heat treatment process of exactly weighed quantities of elements Fe and S [21,24,32,33,34]. However, this method proceeds under complicated conditions such as low vacuum and high temperature and is also time-consuming. Moreover, this method allows, as a rule, to obtain bulk samples, while nanosized particles are of particular interest for some applications. Hydrothermal (solvothermal—developed based on the hydrothermal synthesis, but with using organic solvents) methods have been used for the fabrication of various iron sulfide nanoparticles [14,35,36,37]. However, these methods have a long reaction time and proceed in a sealed environment at high pressures and temperatures [38,39]. In previous works [40,41], the thermal decomposition method has been successfully used to synthesize Fe_1-x_S nanoparticles, however, the described process was carried out in several stages with the preliminary preparation of iron-oleylamine and sulfur-oleylamine complexes, which complicates the preparation of pyrrhotite nanoparticles.

In this study, pyrrhotite nanoparticles, namely Fe_7_S_8_ and Fe_9_S_10_, were synthesized via a simple one-step thermal decomposition method in which hexadecylamine was used as a solvent. Moreover, the process proceeds at atmospheric pressure and does not require sophisticated equipment, making it easily reproducible. The effect of the reaction temperature on the phase formation and morphology of the obtained nanoparticles was investigated, and their magnetic properties were discussed.

## 2. Synthesis and Experimental Techniques

In the present research, Fe_1-x_S samples were synthesized by the thermal pyrolysis method using iron (III) nitrate nonahydrate (Fe(NO_3_)_3_·9H_2_O) (Merck Millipore), sulfur powder (PanReac AppliChem ITW Reagents), and hexadecylamine (Fisher Scientific International, Inc.) (HDA). All the reagents were of analytical grade and were used without any further purification. In a typical synthesis process, 1-hexadecylamine was melted at 80 °C in a three round-bottomed flask, and then Fe(NO_3_)_3_·9H_2_O and sulfur powder were added. The mixture was heated to 120 °C and kept at this temperature for 30 min under magnetic stirring to remove water. At the final stage of the process, the temperature was raised to the reaction temperatures (T_R_), which varied from 360 ≤ T_R_ ≤ 400 °C and kept at the appropriate temperatures for 1 h. To remove HDA, obtained nanoparticles were washed several times with toluene heated to 70 °C.

The phase formation of the nanoparticles obtained has been investigated using a SHIMADZU XRD-600 X-ray diffractometer (Shimadzu Corporation, Japan, Kyoto) (Cu Kα radiation, 40 kV, 30 mA, λ = 1.5418 Å) in the 2θ range 20–80°. The morphology and particle size of the nanoparticles have been characterized using the JEOL JEM-1230 transmission electron microscope (JEOL Ltd., Japan, Tokyo) operated at an accelerating voltage of 120 kV. Magnetic properties have been studied via a vibrating sample magnetometer (Lakeshore 7400 series VSM (Lake Shore Cryotronics Inc., Westerville, OH, USA) in the applied field of H = ± 15 kOe.

## 3. Results and Discussion

### 3.1. XRD Analysis

**Figure 1:**
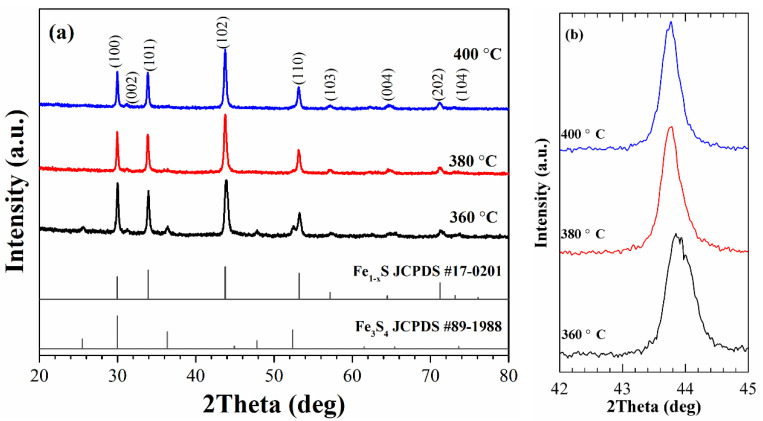
XRD patterns of the as-synthesized samples (**a**) and (102) diffraction peak of the samples in enlarged scale (**b**).

The X-ray diffraction (XRD) patterns of the as-synthesized nanoparticles are shown in Figure 1a. The peaks observed in the XRD patterns of the samples, synthesized at 380 and 400 °C, match well with hexagonal pyrrhotite Fe_1-x_S structure (JCPDS Card N° 17-0201) and do not contain traces of other phases. At the same time, for the sample synthesized at 360 °C, additional weak peaks occur at ~25.5°, 36.3 °, and 52.4 °. These peaks are consistent with JCPDS Card No. 89-1988 of cubic greigite, indicating that the sample contains a small fraction of Fe_3_S_4_.

The identification of phase composition of the synthesized nanoparticles was carried out based on the results obtained in previous studies. Arnold [33,42] has determined a correlation between the Fe/S ratio and the position of (102) peak, which was successfully used [34] to determine the phase composition of Fe_1-x_S powders. In our experiments, the positions of the peak (102) for the samples synthesized at T_R_ = 360, 380, and 400 °C are at 43.91, 43.77, and 43.76°, respectively, and they can be attributed to the following compositions: Fe_7_S_8_ (the sample synthesized at 360 °C) and Fe_9_S_10_ (the samples synthesized at 380 and 400 °C). It should be noted that the XRD pattern of the sample synthesized at 360 °C does not show the splitting of the peak (102), which is an indication of the formation of monoclinic pyrrhotite [26]. Therefore, it can be concluded that the synthesized sample is hexagonal Fe_7_S_8_ and has a 3C superstructure (a = 2A; c = 3C) [20,43]. Thus, the synthesis method allows controlling the phase formation of Fe_1-x_S nanoparticles by changing the reaction temperature.

### 3.2. TEM Data

**Figure 2:**
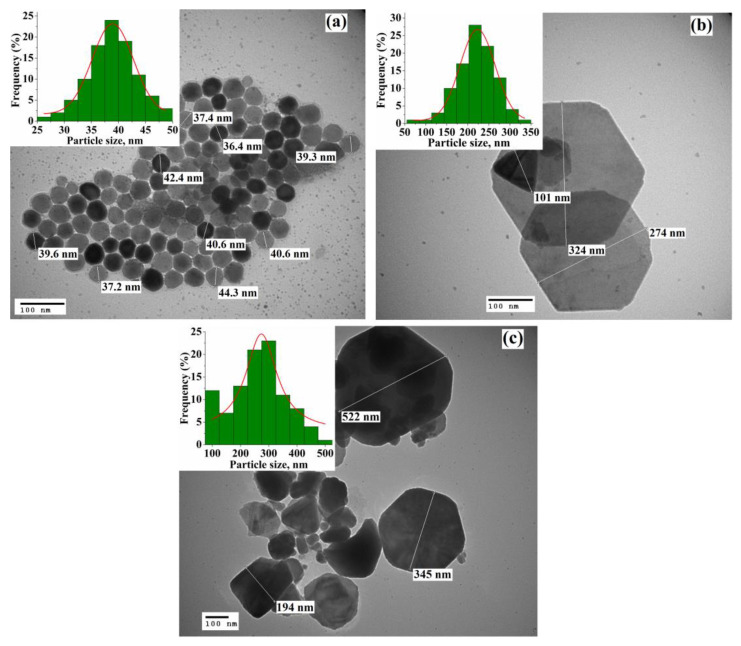
TEM images of the Fe_1-x_S nanoparticles synthesized at (**a**) T_R_ = 360 °C, (**b**) T_R_ = 380 °C, and (**c**) T_R_ = 400 °C. The insets show the particle size distribution for each sample.

The TEM images of the nanoparticles synthesized at 360, 380, and 400 °C are shown in Figure 2. It can be seen that nanoparticles synthesized at 360 °C are almost uniform in size and have a quasi-spherical or hexagonal shape. The particle size of the sample (inset on Figure 1a) oscillates between 25 and 50 nm and the average value obtained by Gaussian fitting is 39 nm. Meanwhile, with an increase in the reaction temperature to 380 and 400 °C, a significant increase in the upper limit of the particle size is observed (to ~350 and 550 nm, respectively). Herewith, the nanoparticles obtained at 380 °C retain accurate shape (hexagonal or triangular) with an average size of 223 nm; however, a further increase in the reaction temperature leads to the formation of mainly disordered in shape nanoparticles with an average size of 274 nm. The sharp increase in the size of Fe_1-x_S nanoparticles is related to an unconstrained dissolution-precipitation (Oswald ripening) process, in which the synthesis temperature significantly affects the growth rate of nanoparticles [44]. With an increase in the reaction temperature, the dissolution rate of small nanoparticles in a supersaturated solution increases, leading to the formation of larger nanoparticles. At the same time, their plate-like shape can be associated [14] with the preferential growth of the low-interfacial energy surface planes.

### 3.3. Magnetic Measurements

**Figure 3:**
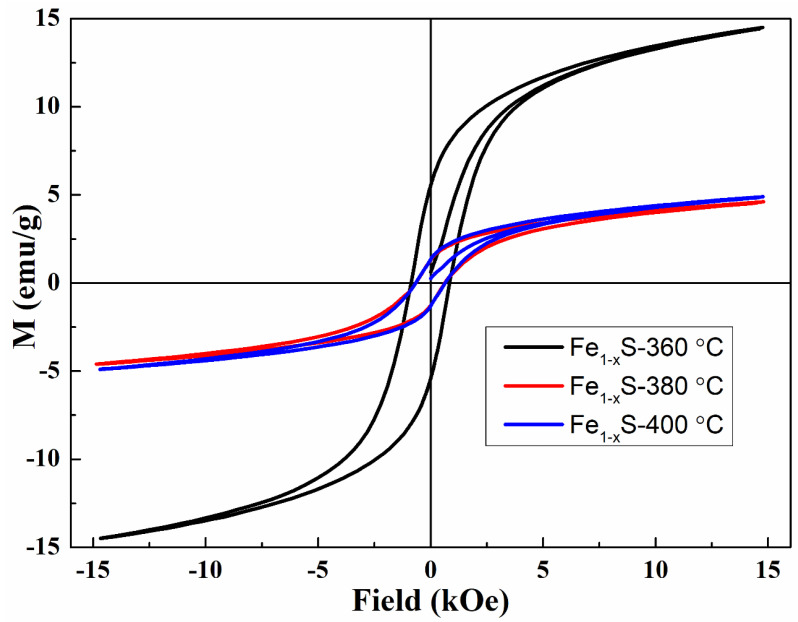
Magnetization curves of the samples synthesized at T_R_ = 360, 380, and 400 °C.

The magnetic hysteresis loops of the synthesized nanoparticles measured at room temperature are shown in Figure 3.

As can be seen, all samples demonstrate ferromagnetic (ferrimagnetic) behavior at room temperature with hysteresis at low fields and with the values of saturation magnetization, coercivity, and remanent magnetization listed in Table 1. The larger saturation magnetization of the Fe_7_S_8_ sample is related to the presence of a larger number of iron vacancies in alternating layers, which leads to an increase in the uncompensated moment and an increase in M_S_. Another reason for the increase in saturation magnetization is the presence of a small fraction of Fe_3_S_4_ identified by XRD measurements and which has a higher value of M_S_. [7,45].

**Figure 4:**
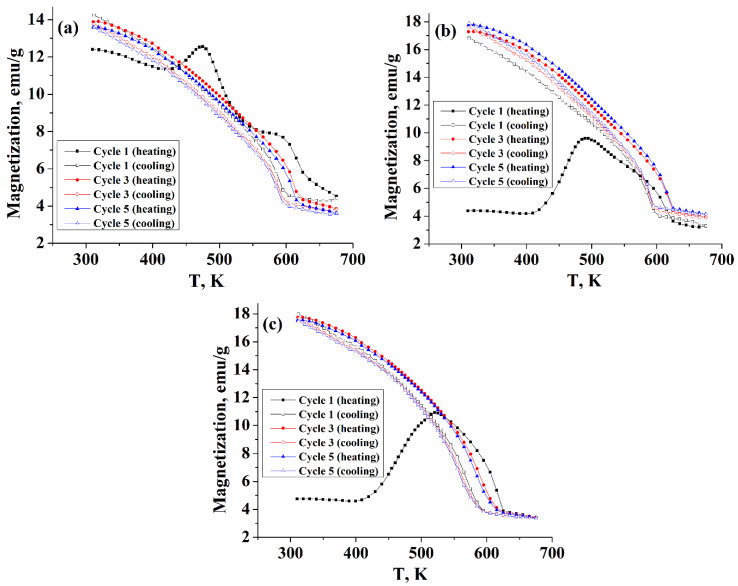
Temperature-dependent magnetization of the nanoparticles obtained at (**a**) T_R_ = 360 °C (4C), (**b**) T_R_ = 380 °C (5C), and (**c**) T_R_ = 400 °C (5C).

Temperature-dependent magnetizations of five repeated heating-cooling cycles obtained at a field of 15 kOe of the synthesized samples are presented in Figure 4 (for better visualization, only cycles 1, 3, and 5 are shown in the figure). In the first heating cycle, the Fe_7_S_8_ sample demonstrates mixed behavior, including λ-peak transition (starting around 425 K and with the maximum at ~475 K) and a Weiss-type component. Besides, near the Curie temperature (in the range of ~565–630 K), there is a slight deviation from the Weiss behavior associated with the presence of a small fraction of greigite in the sample.

However, already in the first cooling branch, the λ-transition is not observed, and the magnetization increases with the Weiss behavior up to room temperature. At the same time, the first heating branches of the samples obtained at T_R_ = 380 and 400 °C demonstrate a behavior different from that of the T_R_ = 360 °C sample. Magnetization started around 4.5 emu/g, consistent with the behavior of magnetic hysteresis loops of these samples. The λ—peak transition is more pronounced and starts at about 405 K with a maximum at 492 K (520 K for the sample with T_R_ = 400 °C), however, during the first cooling branch from 650 K, the peak transition was not reproduced, and magnetization increased with the Weiss behavior to room temperature, herewith the value of magnetization measured in the cooling regime increased significantly. Such changes in the behavior of the magnetization for the samples synthesized at T_R_ = 380 and 400 °C can be associated with the fact that during the first heating branch, a redistribution of vacancies between fully occupied and ordered defective iron layers take place, which leads to a change in the initial phase composition of these samples and a change in their superstructure.

The results of repeated heating/cooling cycles show that in other cycles for all samples, the λ-anomaly is not observed, and the magnetization demonstrates only Weiss-type behavior, herewith the Curie transition on the cooling curves is behind the heating curves by about 30 K. The authors of work [26] have found that for peak-type pyrrhotite with composition Fe_0.906_S, the λ-anomaly is recovered upon cooling the sample, herewith, the process is accompanied by the formation of a Weiss-type component, and such behavior is reproduced in the next heating-cooling cycle. The authors concluded that during heating, the peak-type pyrrhotite (with an antiferromagnetic structure) was partially converted into Weiss-type pyrrhotite (with a ferrimagnetic structure) and that this structure is partially retained upon cooling. However, for the Fe_9_S_10_ samples synthesized in this work (the ordered phase Fe_9_S_10_ corresponds to the composition Fe_0.9_S [21]), the λ-anomaly is not observed both in the first cooling branch and in subsequent heating-cooling cycles, that demonstrate only the Weiss-type behavior, characteristic of ferrimagnetic pyrrhotites, which is consistent with the assumption that an irreversible change in the superstructure of the samples occurs during the first heating branch.

The samples after five heating-cooling cycles were again subjected to XRD analysis to compare the phase compositions before and after high-temperature magnetic measurements. The obtained XRD data after heating/cooling cycles demonstrate that the synthesized samples are stable and do not degrade during the measurements.

**Figure 5:**
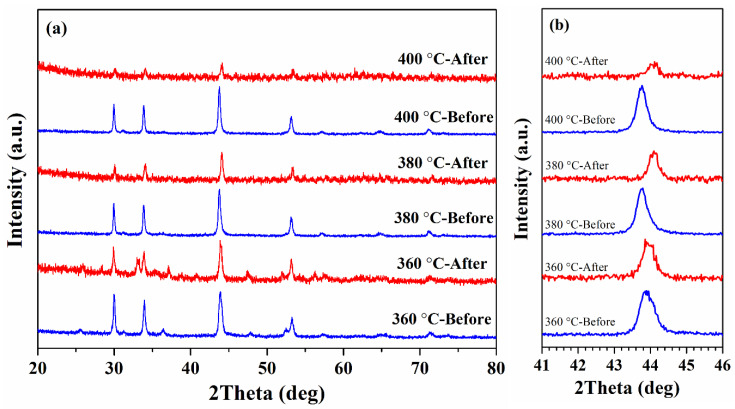
(**a**) XRD patterns of as-synthesized and after five heating-cooling cycles Fe_1-x_S nanoparticles; (**b**) (102) diffraction peak of the samples before and after high-temperature magnetic measurements in enlarged scale.

As can be seen from Figure 5a, in the sample synthesized at 360 °C, except for the original peaks, an unidentified peak appears at 33.1°, while the peak (102) position did not change (Figure 5b). At the same time, for the samples obtained at T_R_ = 380 and 400 °C, a shift of the peak (102) towards larger angles (~44° for both samples) is observed. Thus, it can be concluded that the phase composition of these two samples changed from Fe_9_S_10_ to Fe_7_S_8_ and high-temperature magnetic measurements led to a change in the superstructure of the samples with 5C to 3C.

## 4. Conclusions

A simple thermal decomposition method involving iron (III) nitrate nonahydrate, sulfur powder, and hexadecylamine as a solvent for the synthesis of Fe_1-x_S nanoparticles is presented. The formation of Fe_7_S_8_ (3C superstructure) at T_R_ = 360 °C and Fe_9_S_10_ (5C superstructure) at T_R_ = 380 and 400 °C nanoparticles was confirmed by structural analysis, herewith, it was found that an increase in the reaction temperature leads to a sharp increase in particle size. High-temperature magnetic measurements in five repeated heating-cooling cycles demonstrated that in the first heating branch, the samples obtained at T_R_ = 380 and 400 °C displayed markedly different behavior compared to the sample with T_R_ = 360 °C with a clearly pronounced the λ—Peak transition. However, already during the first cooling branch from 650 K, the peak transition was not reproduced, and magnetization increased with Weiss’s behavior back to room temperature. The observed change in magnetization behavior is explained by a redistribution of vacancies which leads to a change in the initial phase composition, which is in agreement with the results of the structural analysis of the samples after high-temperature magnetic measurements.

## Figures and Tables

**Table 1:** The values of saturation magnetization (M_S_), coercivity (H_C_), and remanent magnetization (M_R_) of the synthesized Fe_1-x_S nanoparticles.

T_R_, °C	M_S_, emu/g	H_C_, Oe	M_R_, emu/g
360	14.7	868	5.5
380	4.6	648	1.3
400	4.9	659	1.35

## Data Availability

The data presented in this study are available on request from the corresponding author. The data are not publicly available due to they also form part of ongoing research.
